# Thoracoscopic radical surgery for a morbidly obese patient with early lung cancer after laparoscopic sleeve gastrectomy: a case report

**DOI:** 10.1186/s40792-020-00950-6

**Published:** 2020-07-31

**Authors:** Shingo Iwata, Akeo Hagiwara, Yutaka Harima

**Affiliations:** 1 Department of Surgery, Takeda General Hospital, 28-1 Ishida, Moriminami-cho, Hushimi-ku, Kyoto, 601-1495 Japan; 2 Faculty of Life and Medical Sciences, Doshisha University, 1-3 Tatara, Miyakodani, Kyotanabe, Kyoto, 610-0394 Japan

**Keywords:** Morbid obesity, Weight reduction before surgery, Laparoscopic sleeve gastrectomy, Bariatric surgery, Lung cancer

## Abstract

**Background:**

We experienced a case of early stage lung cancer involving a morbidly obese patient. Obesity is associated with a higher incidence of surgical complications. We examined the effectiveness of laparoscopic sleeve gastrectomy as a primary weight loss procedure in a morbidly obese patient who required oncological surgery.

**Case presentation:**

A 64-year-old morbidly obese female with a body mass index of 43.5 kg/m^2^ was referred to our hospital to undergo weight loss. A right-sided lung mass was found incidentally on computed tomography conducted in preparation for laparoscopic sleeve gastrectomy, which was performed prior to tumor surgery. As a result, weight loss was achieved within 2.5 months after the laparoscopic sleeve gastrectomy, and the patient’s type-2 diabetes, hypertension, and dyslipidemia, which are linked to obesity, were markedly ameliorated. After a quick intraoperative pathological inspection revealed that the tumor was malignant, thoracoscopic right lung superior lobe resection was performed safely.

**Conclusions:**

Laparoscopic sleeve gastrectomy proved to be a powerful approach in a case in which a morbidly obese patient with early stage cancer needed to lose weight rapidly.

## Background

It is necessary to approach surgery for neoplastic tumors in morbidly obese patients very carefully from the viewpoints of anesthesia, the increased degree of difficulty of the operation, and postoperative complications. Oncological surgery can be performed more safely after the high surgical risk associated with morbid obesity is decreased by weight reduction. When difficulty achieving weight reduction or weight rebound is seen after internal medicine therapy, bariatric surgery is reported to be effective [1–4]. Laparoscopic sleeve gastrectomy (LSG) is considered to be useful for such purposes, as it has a more acceptable profile in terms of the operative time, intraoperative blood loss, and perioperative morbidities [5] than other bariatric surgeries. Few previous studies have discussed the indications for performing LSG in morbidly obese patients as a first step to treating malignancies. Herein, we describe a case in which LSG was effective at achieving weight reduction in a patient whose morbid obesity would have made radical surgery for early stage lung cancer difficult.

## Case presentation

### The patient

The patient was a 64-year-old female.

### Chief complaint

The patient wanted to undergo bariatric surgery.

### Anamnesis

At the age of 20, the patient’s body weight (BW) was 42 kg. Her BW gradually increased after she got married and gave birth, reaching 80 kg at the age of 62, which led her to consult an internist. Weight reduction was attempted. This initially resulted in a 10-kg reduction in her weight, but it subsequently rebounded to over 87 kg. The patient tried conservative weight reduction, but it only resulted in her losing 2 kg. Since the patient’s obesity was resistant to medical treatment and was accompanied by type-2 diabetes mellitus, hypertension, and dyslipidemia, the internist recommended bariatric surgery, and the patient was referred to our hospital.

### History

The patient had suffered a cerebral infarction at the age of 55 years, and right-sided hemiplegia remained. She also had type-2 diabetes mellitus, hypertension, and dyslipidemia.

### Clinical data before the LSG

The patient was recognized to be morbidly obese (height, 149.0 cm; BW, 96.7 kg; body mass index (BMI), 43.5 kg/m^2^). Her hemoglobin A1c (HbA1c) level was 7.9 (Table 1). Endocrine tests excluded secondary obesity. The patient’s electrocardiogram was within the normal range.

**Table 1:** Laboratory data obtained at the patient’s first visit, preoperative day 1, and 2.5 postoperative months

	First visit	Pre-LSG 1	Post-LSG 2.5 months
**WBC/μL**	**5600**	**6300**	**5000**
**Hb (g/dL)**	**13.7**	**13.1**	**12.3**
**Hct (%)**	**43.0**	**38.2**	**35.6**
**Plt (× 10**^**4**^**/μL)**	**23.4**	**25.0**	**23.5**
**TP (g/dL)**	**7.2**	**6.4**	**6.0**
**Alb (g/dL)**	**4.0**	**3.6**	**3.4**
**T-Bil (mg/dL)**	**0.4**	**0.6**	**0.6**
**AST (IU/mL)**	**32**	**26**	**22**
**ALT (IU/mL)**	**37**	**37**	**16**
**ALP (IU/mL)**	**279**	**232**	**167**
**LDH (IU/mL)**	**205**	**179**	**188**
**γ-GTP (IU/mL)**	**77**	**72**	**32**
**BUN (mg/dL)**	**8**	**12**	**10**
**CRE (mg/dL)**	**0.59**	**0.65**	**0.85**
**T-Chol (mg/dL)**	**192**	**179**	**215**
**HDL (mg/dL)**	**69**	**65**	**86**
**LDL (mg/dL)**	**104**	**104**	**91**
**TG (mg/dL)**	**151**	**87**	**98**
**HbA1c [NGSP] (%)**	**7.9**	**6.2**	**5.5**
**CRP (mg/dL)**	**0.20**	**0.71**	**0.06**

*Pre-LSG 1* 1 day before the LSG, *post-LSG 2.5 months*, 2.5 months after the LSG, *LSG* laparoscopic sleeve gastrectomy, *WBC* white blood cells, *Hb* hemoglobin, *Hct* hematocrit, *Plt* platelets, *TP* total protein, *Alb* albumin, *T-Bil* total bilirubin, *AST* aspartate transaminase, *ALT* alanine transaminase, *ALP* alkaline phosphatase, *LDH* lactate dehydrogenase, *γ-GTP* gamma glutamyl transpeptidase, *BUN* blood urea nitrogen, *CRE* creatinine, *T-Chol* total cholesterol, *HDL* high-density lipoprotein cholesterol, *LDL* low-density lipoprotein cholesterol, *TG* triglycerides, *HbA1c* hemoglobin A1c, *NGSP* National Glycohemoglobin Standardization Program, *CRP* C-reactive protein

### Pulmonary function tests

Preoperatively, the patient’s percentage vital capacity was 90.2%, and her percentage forced expiratory volume in 1.0 (sec) (Gaensler) was 80.96%. Postoperatively, her pulmonary function recovered to a percentage vital capacity of 99.1% and a percentage forced expiratory volume in 1.0 (sec) (Gaensler) of 101.9%. Her spirographic data were also improved by the weight reduction achieved before the lung surgery.

### Chest computed tomography

**Fig. 1:**
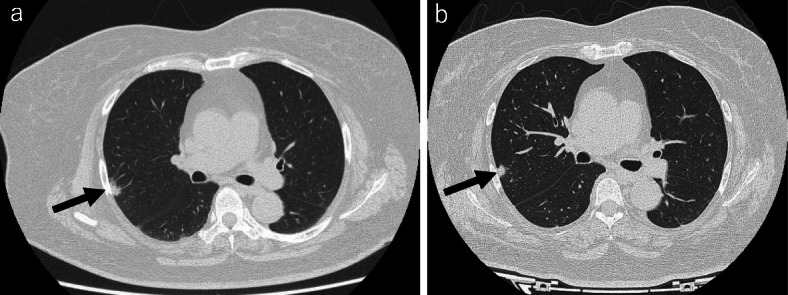
Chest computed tomography scan. **a** An image obtained 1 month before the LSG is shown. A 10-mm mass was observed in the superior lobe of the right lung (arrow). **b** An image obtained 2.5 months after the LSG is shown. No marked change in the size of the lung mass (arrow) was noted, but a reduction in the amount of subcutaneous fat was seen

A nodule shadow was recognized in the right superior lobe of the lung, but it was impossible to determine whether it was an inflammatory or neoplastic lesion (Fig. 1a). No meaningful lymph node swelling was seen in the mediastinum.

## Ethical standards

Before the surgery, written informed consent was obtained from the patient. This study was implemented in compliance with the Helsinki Declaration (as revised in 1975 and 1983) and was approved by the hospital’s ethics committee.

## Clinical course

After adhering to a diet and therapeutic exercise program based on educational admission and outpatient visits, the patient’s BW decreased to 79.8 kg (BMI, 35.9 kg/m^2^), i.e., it decreased by about 17 kg from that seen at her first visit, and her HbA1c level fell to 6.2; therefore, LSG was performed.

**Fig. 2:**
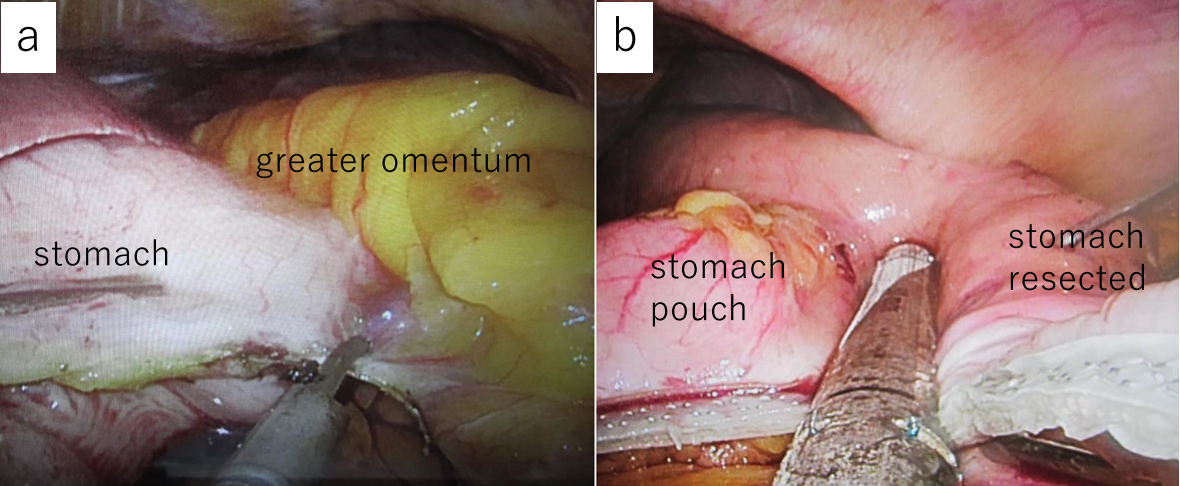
Laparoscopic sleeve gastrectomy. **a** The greater omentum was dissected using a vessel-sealing system from a point located 4 cm from the pyloric ring (on the oral side) to the His angle. **b** The stomach was resected on the side of the greater curvature using a surgical stapling device

**Fig. 3:**
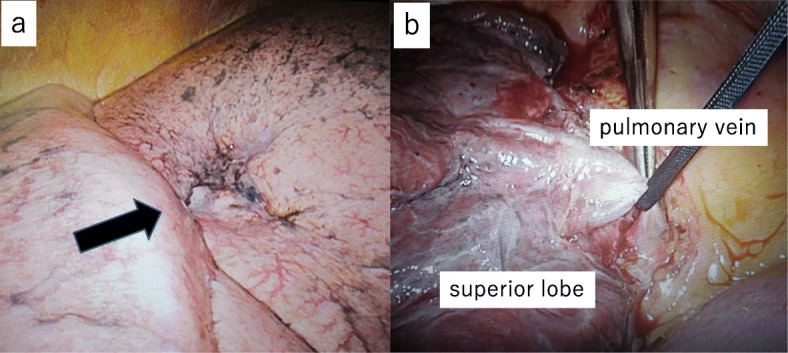
Thoracoscopic surgery. **a** The tumor was located in the superior lobe of the right lung (arrow). **b** Thoracoscopic right lung superior lobe resection was performed

LSG [6]: the procedure, which was performed under general anesthesia, involved longitudinal resection of the greater omentum, starting from the antrum at a point 4 cm from the pylorus along the greater curvature of the stomach and finishing at the His angle (Fig. 2a). After resecting the omentum, the stomach was resected on the side of the greater curvature using a surgical stapling device under endoscopic guidance (Fig. 2b). Thus, after surgically removing a section of the stomach, a small-diameter gastric tube, i.e., a small stomach, was produced. After reinforcing the staple line, endoscopy confirmed that there was no bleeding or contraction within the stomach. The operative time was 3 h and 24 min, and the amount of intraoperative blood loss was 10 mL. No complications were observed after the LSG, and sufficient nutritional guidance was provided. The patient was discharged from hospital 10 days after the surgery. The patient’s type-2 diabetes, dyslipidemia, and hypertension improved without medication after the LSG. Two and a half months after the LSG, her BW and BMI had reduced to 67.2 kg and 30.3 kg/m^2^, respectively, and her HbA1c level had normalized to 5.5% without medication (Table 1). Chest computed tomography showed no marked change in the size of the lung tumor and a reduction in the amount of subcutaneous fat (Fig. 1b). As safe surgery was judged to be possible, a decision was made to resect the lung tumor. Thoracoscopic right lung superior lobe resection was selected, as a quick pathological inspection performed during the operation revealed that the tumor was malignant (Fig. 3a, b). The operative time was 3 h and 42 min, and the amount of intraoperative blood loss was 5 mL. The patient was discharged from hospital 12 days after surgery with no complications. The tumor measured 12 × 10 × 6 mm. Papillary adenocarcinoma was detected during a pathological examination of the resected specimens. No metastases were found in the regional lymph nodes. The TNM stage was pl0, ly0, v0, br (-), PN0, sT1bN0M0 stage IA2.

## Discussion

Recently, along with the adoption of a westernized diet in Japan, the incidence rates of morbid obesity and metabolic syndrome have increased and become serious problems. Obesity itself not only leads to various problems in medical practice due to its physical effects, such as the presence of abundant amounts of visceral and subcutaneous fat, but also causes various related diseases, including diabetes, hypertension, and dyslipidemia. These obesity-related conditions are known to induce postoperative complications, i.e., infections, delayed wound healing, deep vein thrombosis, and pulmonary thromboemboli [7–9]. In morbidly obese patients, since insulin resistance and hyperglycemia inhibit the functions of white blood cells, and hence, weaken immune functions and vascular tissue, surgical wounds can cause many complications [10–14]. Therefore, it is considered that in cases involving morbidly obese patients, surgical operations should be performed after weight reduction, as this results in fewer postoperative complications.

We often see cases in which medical interventions against obesity, including dietary and physical therapy, are not effective or the effects do not last long even though the patient achieves a transient weight reduction. On the other hand, bariatric surgery is a reliable and powerful weight reduction method [1–4].

Motivation is important for treating obesity. In the present case, the patient had a clear purpose for losing weight, i.e., to be able to undergo bariatric surgery and focused on achieving steady preoperative weight reduction within a short period of time. Patients that can decrease their BW are indicated for bariatric surgery because it is judged that they would be able to withstand more severe postoperative diet control. To conduct bariatric surgery safely, it is important to reduce the quantity of visceral fat so that the lateral segment of the liver, which undergoes compensatory hypertrophy in the presence of fatty liver, can be reduced in size as much as possible. In the current case, the patient lost 17 kg (BW, 79.8 kg) of weight before the LSG and 30 kg (BMI, 30 kg/m^2^) after the LSG.

We will now discuss why the patient did not undergo pulmonary surgery when she weighed 79.8 kg. A lung shadow was found incidentally during the course of preoperative weight reduction for bariatric surgery. The radiologist could not determine whether it was malignant, but suggested close follow-up and recommended that chest CT be performed every month. Monthly CT revealed no marked changes in the lung shadow before the LSG. We considered that even if the lesion was malignant, it was probably a very early stage lesion, and therefore, it would be possible to perform a curative operation and achieve a good prognosis 2.5 months later. Although the patient’s BW was 79.8 kg, her BMI was 35.9 kg/m^2^, which was still high, and she had diabetes. In patients with BMI of > 35 kg/m^2^, thoracoscopic surgery for lung cancer carries an increased risk of postoperative complications and mortality. In addition, according to the American Diabetes Association [15], bariatric surgery should be recommended for patients with BMI of 35–39.9 kg/m^2^ who do not achieve durable weight loss or improvements in their comorbidities with non-surgical methods. Our patient’s weight had rebounded over and over again. If bariatric surgery had not been conducted at this point, we expect that her weight would have rebounded again. The purpose of bariatric surgery is not only to reduce the patient’s BW, but also to change their body in a way that helps to prevent their weight rebounding. Bariatric surgery is more effective against obesity than conventional medical therapy in terms of the magnitude of the long-term (20-year period) weight loss achieved [4]. Schauer [16] also reported that patients in their surgical group achieved greater percentage reductions in BW than those in the medical therapy group. In addition, the rebound rate was higher in the medical therapy alone group. The use of glucose-lowering medications and quality-of-life measures also produced more favorable results in the surgical group than in the medical therapy alone group. Therefore, bariatric surgery was required at this point in order to achieve long-term diabetes remission and maintain the weight loss produced by surgery.

LSG appears to be a more suitable procedure because it results in faster weight loss than the use of an intragastric balloon or laparoscopic adjustable gastric banding; involves a simple procedure and a shorter operating time; and causes fewer postoperative complications than bypass surgery, such as laparoscopic Roux-en-Y gastric bypass [5]. LSG might be superior to various other bariatric surgical procedures in terms of its ability to shorten the interval between diagnosis and curative treatment for malignant tumors in morbidly obese patients. Actually, in our case lung surgery could be performed safely within 3 months after the LSG and did not result in any postoperative complications.

Many studies have recommended that orthopedic surgery for orthopedic disease, such as knee osteoarthritis, should be performed after weight reduction in cases involving morbidly obese patients, as this approach results in functional improvements and fewer postoperative complications [17–20]. However, to the best of our knowledge, there are few reports about cases in which bariatric surgery was conducted for the purpose of safely performing cancer surgery. Gianos et al. [21] reported that LSG was performed for the purpose of allowing cancer surgery to be conducted safely in morbidly obese patients with early stage malignancies. The latter study included one case of small bowel carcinoid, two cases of renal tumors, and one case of prostate cancer. In a case reported by Togawa et al. [22], LSG was successful at allowing radical surgery for advanced colon cancer, the progression of which was well controlled by chemotherapy, to be safely performed in a morbidly obese patient. Our two-step method is limited to curable malignancies that are detected at an early stage. Patients with early stage cancer or certain types of cancer, such as those for which chemotherapy would make curative surgery possible, might benefit from this two-stage procedure.

It is known that in obese patients, the risks of various cancers are high [23, 24]. Therefore, the number of cancer patients complicated with morbid obesity might increase along with the increase in the number of obese patients. LSG is a powerful approach for cases in which urgent and assured weight reduction is required in order to allow malignant tumors to be treated. However, further research will be needed to confirm this, and it will be necessary to accumulate a greater number of cases.

## Conclusions

LSG was successfully conducted in a morbidly obese patient in order to allow safe and radical surgery for lung cancer. As a result of the successful preoperative weight reduction, it became possible to safely conduct thoracoscopic lung cancer surgery. LSG is useful for morbidly obese patients with early stage malignancies.

## Data Availability

The datasets supporting the conclusions of this article are included within the article.

## Abbreviations

LSG: Laparoscopic sleeve gastrectomy
BW: Body weight
BMI: Body mass index
HbA1c: Hemoglobin A1c
